# O9.7. INDIVIDUALIZED LONG-TERM OUTCOME PREDICTION OF PSYCHOSIS IN AN OBSERVATIONAL STUDY: A MACHINE LEARNING APPROACH

**DOI:** 10.1093/schbul/sby015.251

**Published:** 2018-04-01

**Authors:** Jessica De Nijs, Daniel P J van Opstal, Ronald J Janssen, Wiepke Cahn, Hugo Schnack, GROUP Investigators

**Affiliations:** 1 Brain Centre Rudolf Magnus, University Medical Center Utrecht; 2 University Medical Centre Utrecht

## Abstract

**Background:**

Schizophrenia and related disorders have heterogeneous outcomes. Predicting long-term psychosis outcome may be helpful in improving treatment decision making. The aim of our study was to develop and validate a long-term outcome prediction model of psychosis in individual patients. Many studies have shown that outcome is related to symptoms, demographic, clinical, cognitive, genetic and environmental data – at the level of correlations. We hypothesized that, using machine learning (ML), it is possible to predict individual long-term outcome based on patterns that are present in these data at baseline. Second, we test if variables that were recently found to be predictive of short-term outcome (European First Episode Schizophrenia Trial (EUFEST), Koutsouleris et al, 2016) can yield accurate long-term outcome predictions in our sample.

**Methods:**

This study included 523 patients (mean (SD) age = 27.6 (7.4) year) from the Genetic Risk and Outcome of Psychosis study. The study extensively assessed patients at baseline, 3- and 6-year follow-up. Outcome was defined in two ways: 1) Symptomatic: being in remission (good outcome) or not in remission (poor outcome), according to the Remission Tool (i.e. a consensus definition which defines remission as maintaining core DSM symptoms, based on Positive and Negative Symptom Scale [PANSS] on a low level during ≥6 months); and 2) Functional, using Global Assessment of Functioning (GAF) scale, divided into good (GAF≥65) and poor (GAF <65) outcome. A support vector machine was trained to predict outcome based on (combinations of) the following sets of baseline data: PANSS, clinical and demographic variables, substance use, neurocognitive/ social cognitive tasks, premorbid adjustment, need of care items (CANSAS), extrapyramidal symptoms, genetic features, environmental variables; and the sets of predictors from 4- and 52-week GAF-based outcome prediction models from the EUFEST study. We trained full and leaner models, using recursive feature elimination (RFE). We tested performance of outcome prediction models using nested cross-validation, i.e., predicting outcome in patients not part of the training set.

**Results:**

6-year functional outcome (i.e. GAF status) was best predicted by a multi-modal model based on baseline PANSS, CANSAS, clinical and demographic variables, using RFE: 75% of the patients was correctly predicted. Significant predictions using single-modal models were obtained for baseline PANSS (62.7%), clinical (60.9%) and CANSAS predictors (58.0%). For functional outcome (GAF) at 6 years, also baseline PANSS, clinical and CANSAS related features produced highest accuracies (61.1%, 63.1% and 59.3% resp.). Classification of symptomatic and functional outcome at 3 years yielded comparable results. Replication using the best scoring predictors of 4 and 52 weeks outcome in the EUFEST study resulted in accuracies of 61.5% and 56.5% for remission 3-year outcome; 61.6% and 61.0% for remission 6-year outcome; 60.1% and 57.7% for GAF 3-year outcome; 62.3% and 64.6% for GAF 6-year outcome.

**Discussion:**

Our results show that predicting long-term symptomatic and functional outcome can be done with reasonable accuracies of up to 75%. Training a ML algorithm revealed that PANSS, clinical and need of care features predicted our multiple endpoints best. Interestingly, EUFEST predictors included these three types of data as a main part of best performing predictors. We showed that these short-term outcome predictors are, to certain extent (up to 65%), also predictive of long-term outcome. Our study is a promising step in pursuit of personalized medicine applicability in mental care institutes. However, our model needs replication in independent samples.

